# Impact of climate change on the integrity of the superstructure of deteriorated U.S. bridges

**DOI:** 10.1371/journal.pone.0223307

**Published:** 2019-10-23

**Authors:** Susan Palu, Hussam Mahmoud

**Affiliations:** Department of Civil and Environmental Engineering, Colorado State University, Fort Collins, Colorado, United States of America; Universita degli Studi di Napoli Federico II, ITALY

## Abstract

Bridges in America are aging and deteriorating, causing substantial financial strain on federal resources and tax payers’ money. Of the various deterioration issues in bridges, one of the most common and costly is malfunctioning of expansion joints, connecting two bridge spans, due to accumulation of debris and dirt in the joint. Although expansion joints are small components of bridges’ superstructure, their malfunction can result in major structural problems and when coupled with thermal stresses, the demand on the structural elements could be further amplified. Intuitively, these additional demands are expected to even worsen if one considers potential future temperature rise due to climate change. Indeed, it has been speculated that climate change is likely to have negative effect on bridges worldwide. However, to date there has been no serious attempts to quantify this effect on a larger spatial scale with no studies pertaining to the integrity of the main load carrying girders. In this study, we attempt to quantify the effect of clogged joints and climate change on failure of the superstructure of a class of steel bridges around the U.S. We surprisingly find that potentially most of the main load carrying girders, in the analyzed bridges, could reach their ultimate capacity when subjected to service load and future climate changes. We further discover that out of nine U.S. regions, the most vulnerable bridges, in a descending order, are those located in the Northern Rockies & Plains, Northwest and Upper Midwest. Ultimately, this study proposes an approach to establish a priority order of bridge maintenance and repair to manage limited funding among a vast inventory in an era of climate change.

## Introduction

It is not a surprise that infrastructure in America and other countries around the world is aging and deteriorating, as a result in increase in demand due to population growth and limitation in resources required for proper inspection and maintenance. According to the National Academy of Engineering, the urban infrastructure restoration and improvement is ranked among the greatest challenges for the Engineers of the 21st century [1]. In order to keep track of the infrastructure condition in the U.S., every four years the American Society of Civil Engineers (ASCE) evaluates sixteen fundamental categories of the U.S. infrastructure such as energy, drinking water, schools, roads, dams, bridges, among others, and issues a report card, assigning a grade to each category based on the physical conditions and investments needed for improvement [2]. In 2017, bridges in the U.S. received a grade C+ [2], as a reflection of their current condition. Undeniably, since the first report card was issued in 1998 the grade for U.S. bridges has been incrementally increasing but hovering around the C range for the last twenty years [3].

Historically, since the collapse of the Silver Bridge over the Ohio River in December of 1967, which resulted in 46 casualties, more attention has been given to establish sound procedures for inspection and management of U.S. bridges [4,5]. The collapse that occurred during the rush hour was attributed to failure of a structural element and in part due to poor inspection [4,5]. As a consequence of that catastrophe, the Federal-Aid Highway Act of 1968 created the National Bridge Inspection Program and established a unified bridge proper safety inspection standard [6,7]. Despite such effort, in 1983 the Mianus River Bridge in Greenwich, Connecticut, collapsed, due to insufficient maintenance, causing three fatalities and resulting in more stringent regulations regarding inspections and safety of bridges [6].

**Fig 1:**
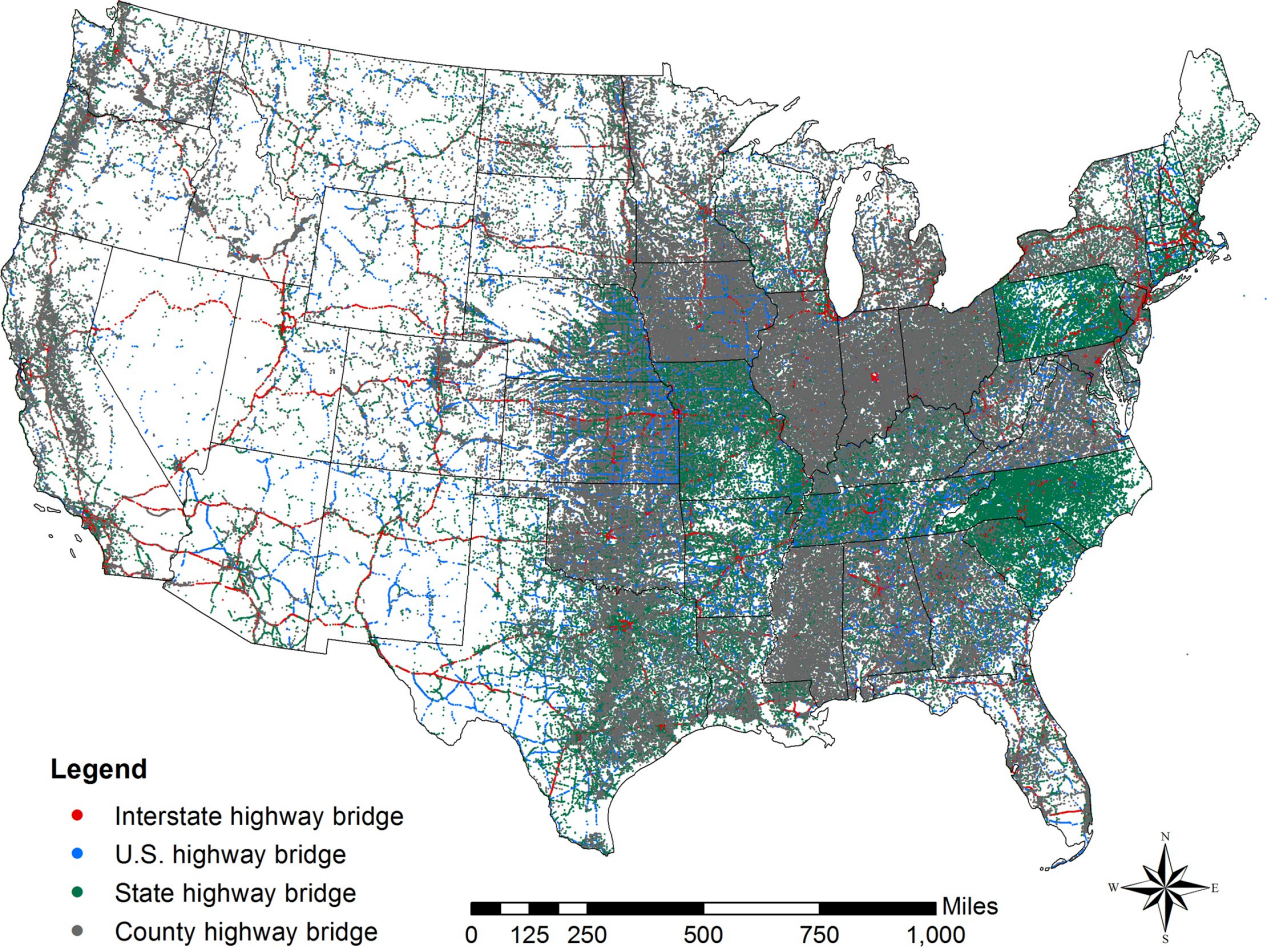
Highways bridges in the United States.

Since 1968, the Federal Highway Administration (FHWA) has developed and maintained the National Bridge Inventory (NBI)–a substantial database that currently contains comprehensive information of every bridge longer than 6 m (20 feet) on all public roads. The inventory is annually updated with the aim of guaranteeing public safety through identification and evaluation of bridge deficiencies [7]. According to NBI 2017 [8], the United States possess 615,002 highway bridges. These bridges are part of the National Highway System comprising of 76,564 km (47,575 miles) of Interstate Highways plus 289,119 km (179,650 miles) of major roads, which carries most of the highway passenger traffic and freight in the U.S. [9]. Fig 1 shows the distribution of every highway bridge in the U.S. The cartographic boundary of U.S. was obtained from the United States Census Bureau [10].

**Fig 2:**
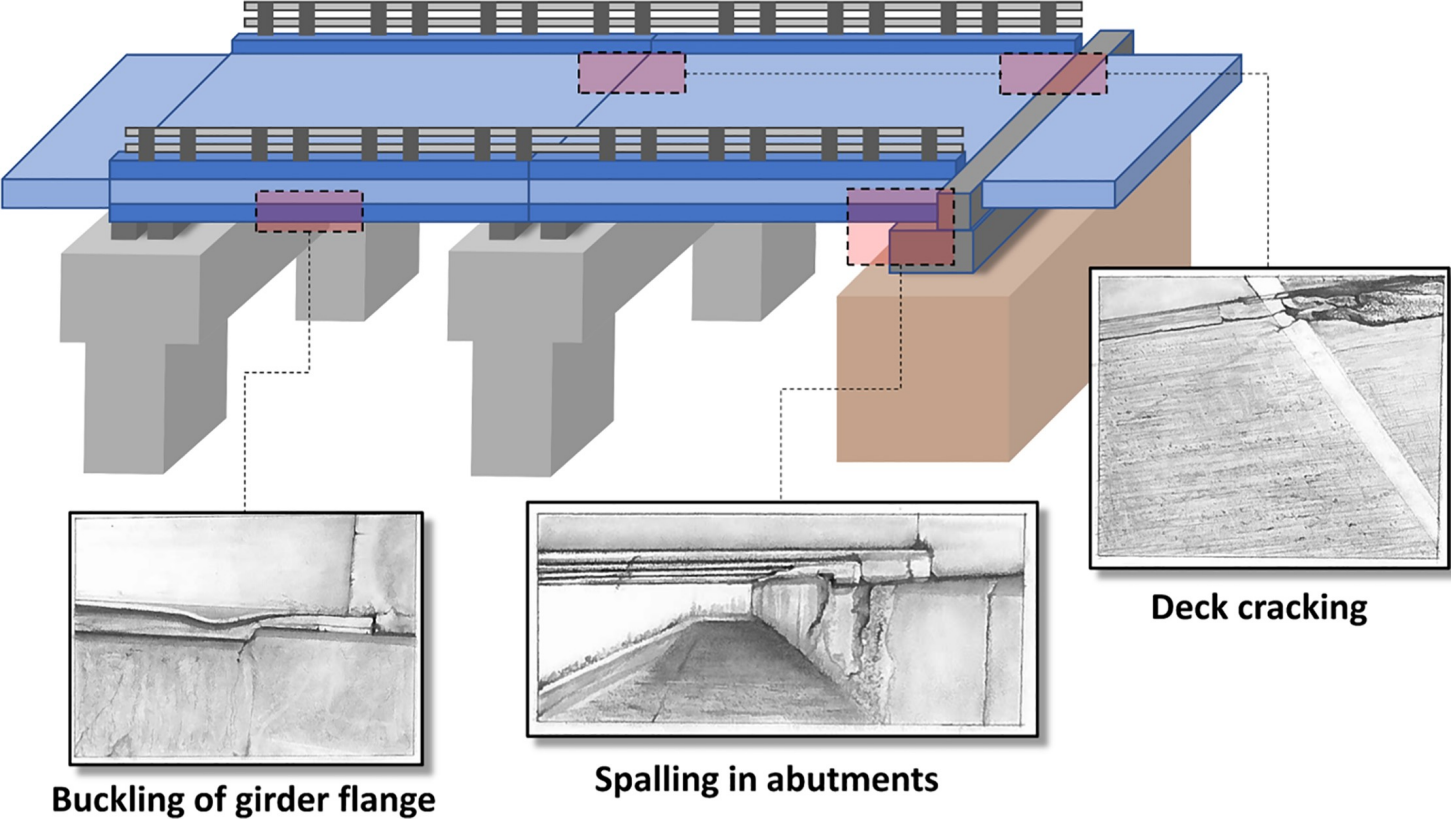
Potential bridge damages caused by the combination of clogged joint condition and unpredicted thermal stresses.

The 2017 NBI reveals that four in ten bridges are 50 years or older, reaching or even exceeding their design life with the average age of bridges in America being 45 years old [8]. In addition, it is noted that 54,560 bridges in U.S. were characterized as being structurally deficient [8] where ‘deficient’ implies that elements of the bridge structure were found in poor conditions due to deterioration or damage [2]. Despite the poor conditions of these bridges, there were approximately 188 million trips across them each day in 2016 [2]. Undoubtedly large spending is required to address a host of deterioration issues in these bridges. These issues include clogging of expansion joints with road debris, scour of foundations caused by water flow, corrosion of structural elements and components due to improper drainage or leakage through damaged expansion joints, deck deterioration due to standing water and deicers, decay or misalignment of bearings, cracks in bases due to uneven settling of foundation, among others [4]. It is important to note that the effect of these listed problems on bridge performance will vary in terms of their level of impact. For example, deck deterioration is expected to cause traffic delay while large scour could threaten the integrity of the structure. Of course, it is important to acknowledge that the criticality of each of these problems might change due to their coupling nature. In this study, we chose to focus on expansion joints in bridges. This is because even though innumerable components of the bridges throughout the country require maintenance or replacement, the deterioration of bridge deck expansion joints, is one of the most common issues [11]. In spite of being small components, if expansion joints do not perform properly, it can affect major structural elements of a bridge [12]. This problem becomes even more significant given the abundance of deck joints bridges in the country. Such frequency is the result of the widespread adoption of simply supported spans design type, which facilitated the construction of a large quantity of roadways in U.S. after the 1956 Federal-Aid Highway Act. However, at that time, potential issues and costs associated with maintenance of deck joints were overlooked [13,14]. As such, the maintenance cost to keep expansion joints clean and functional has been a burden to the American transportation agencies [15]. Fig 2 shows what is typically known as major potential damages to structural elements of simply supported bridges due to the combination of clogged joints condition and unpredicted thermal stresses. These include local buckling of the main girder flanges, spalling of concrete of the abutments, and cracking and crushing of the roadway deck. Some of these issues could arguably fall under the category of serviceability limits. Meaning, local buckling for example would not compromise the safety of the structure. In this study we focus on what is typically an overlooked but critical issue. That is to evaluate the effect of joint clogging on increasing the demand on bridge girders and the deck slab which are the main load carrying elements of the superstructure. The criticality of evaluating this criterion stems from the fact that failure of the girders and crushing in the concrete deck could simply compromise the functionality and structural integrity of the bridge. The integrity, or lack of residual capacity, will depend on the level of redundancy in the bridge and the presence of alternative load path. That is to say failure of the girders and crushing of the concrete may or may not present major safety concerns; however, immediate actions should be taken.

Expansion joints are transversal spaced gaps along the bridge length, with the purpose of allowing longitudinal movements of expansion and contraction of the superstructure when it is subjected to a temperature variation. Nevertheless, they easily become clogged with debris, preventing the bridge from expanding when it is exposed to a temperature rise. In addition, deteriorated joints allow debris, water and deicing salts to infiltrate underneath the bridge deck and accumulate where the bearings are located. This could cause the sliding bearings, which are components that transfer the loads from the superstructure to the substructure of the bridge and allow thermal movement of the girders as well, to corrode and lock up, further preventing the bridge from accommodating thermal movements [12]. As a result, unpredicted thermal stresses for which elements of the bridge were not designed for are ultimately imposed on the superstructure [13,14,16]. This effect can even be amplified considering the projected future temperature changes [17]. According to the recently released Climate Change Adaption Guide for Transportation Systems Management Operations and Maintenance (U.S Department of Transportation–Federal Highway Administration), bridges with joints are more susceptible to damage due to their sensitivity to temperature [18]. Therefore, more attention should be given for these joints since change in climate may require different maintenance and rehabilitation approaches. This was further highlighted by the ASCE Committee on Adaptation to a Changing Climate who not only noted the importance of adapting transportation infrastructure to a changing climate but also emphasized that a changing climate may affect bridge expansion joints [19].

**Fig 3:**
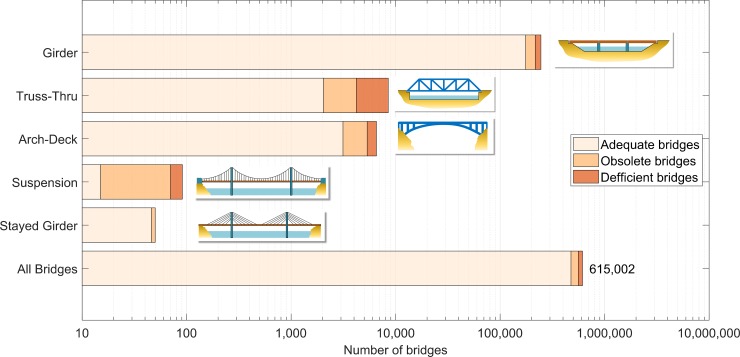
Distribution of bridges built in USA by type of design (number of bridges in log-scale).

One should note that the vulnerability of infrastructure in general to climate change has recently become a topic of debate and research among engineers, researchers, and policy makers [20–28]. Here, we evaluate the vulnerability of 89,089 simply supported steel girder bridges (hereinafter SSSG bridges) in the U.S. due to clogged joints and climate change effects by focusing on the capacity of the main load carrying girders. The superstructure of the 89,089 bridges analyzed comprise of steel-concrete composite sections (see Materials and methods). The NBI database classifies bridges by the type of material they were built with–steel, concrete, wood, masonry as well as the type of their structural design such as girder, truss, arch, suspension, stayed, box girder, among others [29]. The girder type, which consists of two or more longitudinal beams that span over the piers to support the superstructure weight and the traffic load, is by far the most common design type of bridges built in U.S. as shown in Fig 3. For instance, among the Interstate highway bridges, which carry the largest volume of the nation’s traffic, the girder bridge type corresponds to approximately 60% (see S1 Appendix). We acknowledge that other types of steel bridges are configured with expansion joints and in such case clogging of the joints in these bridges could give rise to global as well as local problems. This could include for example rib-to-deck joints in orthotropic bridge systems [30–32]; however, these types of bridges were not the focus of this study.

Girder-type bridges are highlighted not only by their vast number compared to the other types of bridge design, but also by the fact that more than 50% of all deficient bridges in the U.S. are girder type, with the majority of them being SSSG [8] (see S1 Appendix). The total number of SSSG bridges in the U.S. is 97,393 [8] and the average age of this kind of bridge is 50 years old, surpassing the national average bridge age of 45 years (see S1 Appendix).

**Fig 4:**
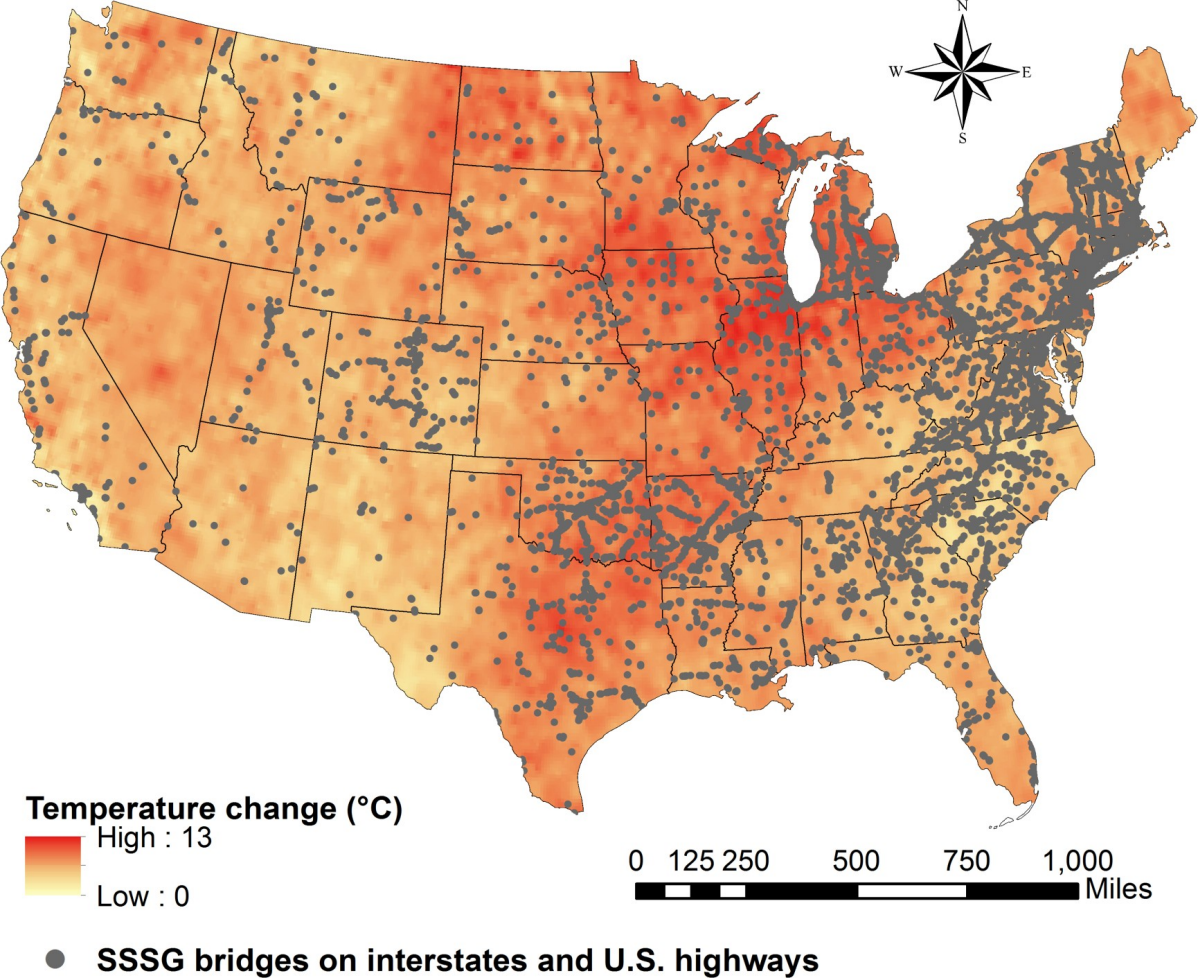
SSSG bridges on Interstate and US highways and projected daily maximum temperature change from 2020 to 2100 under the higher forcing scenario RCP 8.5.

In this study, we evaluate the vulnerability of SSSG bridges in the U.S. under the combined effect of clogged joints and projected temperature rise for 2040, 2060, 2080 and 2100, considering the climate model from the NOAA’s (National Oceanic Atmospheric Administration) Geophysical Fluid Dynamics Laboratory GFDL-CM3 under three Representative Concentration Pathway (RCP) scenarios, which are named for the approximate radiative forcing in year 2100: the lower forcing scenario RCP 2.6, a moderate scenario RCP 6.0 and the higher forcing scenario RCP 8.5 [33] (see Materials and methods and S1 Appendix). One should be aware that this study does not intent to examine all the existing climate models in order to exhaustively minimize uncertainties for future temperatures prediction. Instead, the present study correlates potential temperature rising with the vulnerability of infrastructure. Ultimately, it aims to offer insights on vulnerability of a massive bridge inventory so that management policies can be devised. Fig 4 shows the variation of the projected daily maximum temperature from 2020 to 2100 as well as the location of SSSG bridges that belongs to interstates and U.S. highways based on the mentioned climate model and the higher forcing scenario RCP 8.5.

As one can note, for this particular climate scenario RCP 8.5, the augment of daily maximum temperature along the future years is projected to occur with more intensity in the climate regions: Northern Rockies & Plains, Upper Midwest, Ohio Valley and South. Nevertheless, the amount of thermal stresses developed into the bridge superstructure when expansion joints are clogged do not depend on those future temperatures only, but also on the temperature at the time of construction of the bridge. In other words, the induced thermal stress is a function of the temperature variation and not of the absolute temperature value for which the bridge is subjected. This temperature variation is calculated as the difference between the future daily maximum temperatures projected by the climate model (2040, 2060, 2080 and 2100) and the temperature of the bridge at the time of construction (see Materials and methods). Since the only available information in 2017 NBI [8] is the year in which construction was concluded, the temperature during bridge construction at the stage of expansion joints installation is considered according to four possible scenarios: average of minimum temperatures in the winter (Scenario 1), spring (Scenario 2), summer (Scenario 3) and fall (Scenario 4) for the respective year of each bridge construction conclusion. Each scenario can be interpreted as a hypothetical temperature condition, where Scenario 1 (winter) is the worst case, Scenario 3 (summer) is the most optimistic and Scenarios 2 and 4 present intermediate ranges of temperature (see Materials and methods and S1 Appendix). In addition, bridges are classified to belong to one of the nine U.S. climate regions: Northwest, Northern Rockies & Plains, Upper Midwest, Ohio Valley, Northeast, West, Southwest, South and Southeast (see S1 Appendix). Using this classification, the average of minimum temperatures of each season scenario is taken from the historical NOAA Regional Time Series [34] and attributed to each bridge in the inventory according to its respective year of construction and its regional location (see S1 Appendix). Hence, the temperature range that each bridge is subjected to depends on of the year in which it was built, its geographical location and the future projected temperature.

The structural performance of the SSSG bridges is assessed in terms of the interaction equation, which has long been recognized as design limit state and allows for the quantification of the demand-to-capacity ratio [35]. It is critical to emphasize that this interaction equation, defining the ultimate limit state, is used for structural elements subjected to simultaneous action of bending moments and axial loads. *Bridge girders are only designed for bending moments*. *However*, *under the condition of clogged joints*, *the girders would be subjected to bending moments (due to dead and traffic load) concomitantly with thermal axial load (due to expansion restriction)*. In design codes and guidelines, adequate structural performance and capacity assessment of any element subjected to axial and flexure load combined is ensured by limiting the demand-to-capacity ratio to a maximum value of unity; otherwise, the girder reaches its ultimate load carrying capacity. *The ramifications of the interaction equation exceeding a value of unity will depend on the level of exceedance*. *This could include substantial deformations in the bridge girders*, *slab concrete crushing (in case of composite sections)*, *and subsequent failures in other main load-carrying elements and secondary elements* [36]. *In other words*, *exceeding a value of unity for this equation will render the bridge as failed and immediate actions would have to be taken since it is no longer a serviceability issue but rather an ultimate limit state one*. We note that other issues pertaining to the influence of elevated temperature on durability and potentially the strength of the concrete might be relevant. For example, in a recent study it was shown that environmental temperature, CO_2_ concentration, and a certain range of relative humidity play an important role in the concrete carbonation rates [37], which could be an issue for the concrete decks of the SSSG bridges. However, it was found that even in extreme natural high temperature, the compressive strength and splitting tensile strength is not affected significantly [37].

## Results

**Fig 5:**
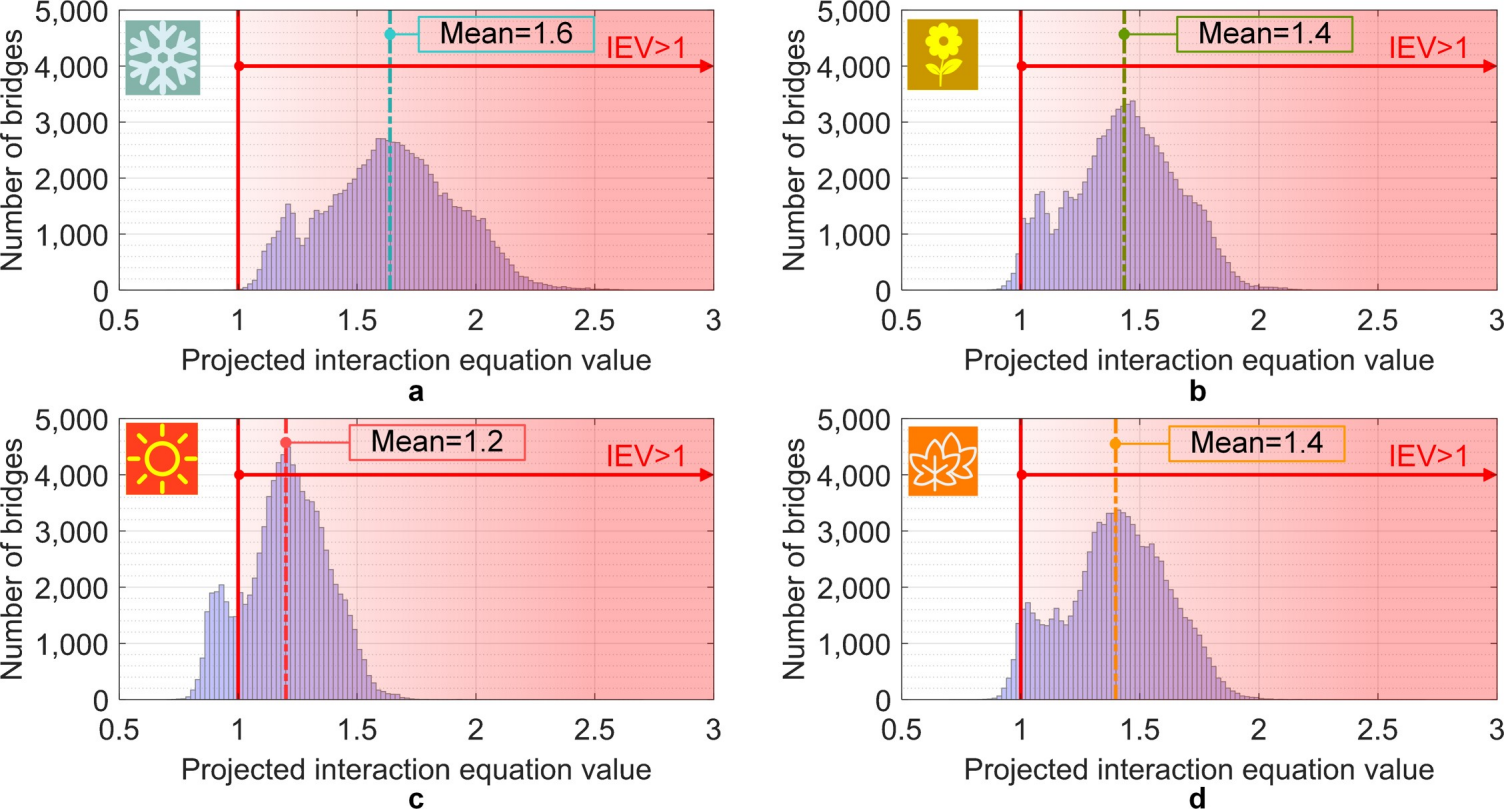
Histograms of the projected interaction equation value (IEV) for temperatures in 2100 under RCP 8.5 considering a) Scenario 1, b) Scenario 2, c) Scenario 3 and d) Scenario 4.

Initially, a sensitivity analysis is carried out to verify the influence of seasonal temperatures during the bridges’ construction. The analysis for the projected temperatures in 2100 (long-term analysis), under the higher forcing climate scenario RCP 8.5 shows that the interaction equation value (IEV) exceeds one for 100% of the SSSG bridges in the U.S., when subjected to the most severe Scenario 1 (assumption of bridges being built during the winter). When subjected to Scenario 2 (spring), the IEV exceeds one for 97% of the bridges. For Scenario 3 (summer), the IEV exceeds one for 83%. Finally, for Scenario 4 (fall), the IEV of one is exceeded for 95% of the bridges analyzed. This is presented in the histograms of Fig 5. Those percentages indicate the amount of bridges in which the demand-to-capacity ratio would be beyond the structural design limits, if no intervention is made. The IEVs shown in Fig 5 are based on the assumption that the accumulated debris between the joints comprises of a mix of gravel and sand. These values are, therefore, expected to change if different material is used in the analysis as shown in Table 1. In addition, the adoption of different RCP’s (2.6 and 6.0) results in slightly smaller values, however following the same presented trend. The pinned case shown in the table reflects a severe condition where the girders are completely restricted from expansion. The severity of the violation of the interaction equation is reduced if a calibrated equation is utilized as shown in Figure I in S1 Appendix. The modified/calibrated equation is based on a recent study in which composite sections were tested under combined axial compression and bending moment [36]. In this case the analysis for the projected temperatures in 2100 shows that the IEV for 63% of the SSSG bridges in the U.S. exceed one for the most severe Scenario 1 (assumption of bridges being built during the winter), followed by 25%, 0.4% and 19% for Scenario 2 (spring), Scenario 3 (summer) and Scenario 4 (fall), respectively. Thus, the hypothesis of bridge construction during winter or summer can be used to represent conservative or extreme scenarios, while the hypothesis of construction during the spring or fall is appropriate for a more general and intermediate analysis.

**Table 1:** Variation of interaction equation value as function of type of debris clogging the joint.

Season	Pinned	Gravel	Mix of Gravel and Sand	Sand
winter	2.2	1.8	1.6	1.4
spring	1.8	1.5	1.4	1.2
summer	1.5	1.3	1.2	1.0
fall	1.8	1.5	1.4	1.2

**Fig 6:**
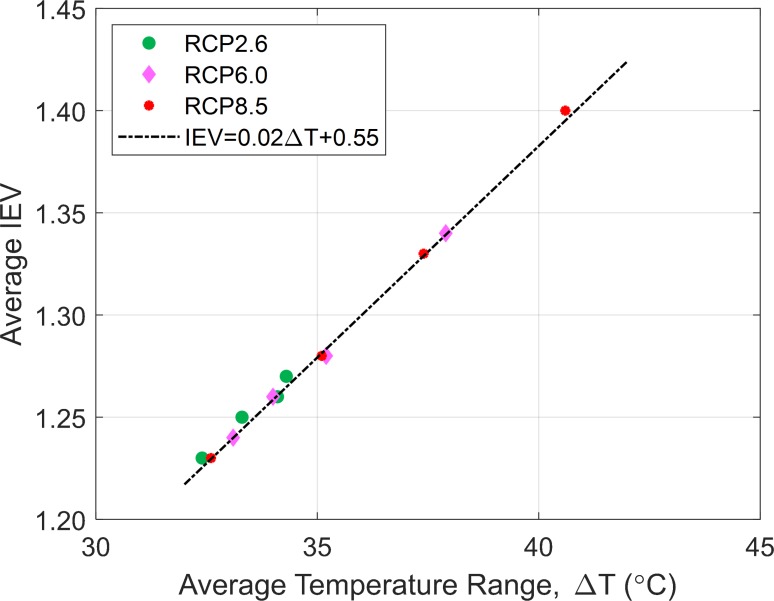
Average IEV as function of average temperature range.

The plot of the average IEV as function of average temperature range for all bridges under analysis (difference between the future temperature and the base temperature of bridge construction during the fall) provides a linear relationship, as illustrated in Fig 6. Specifically, an interesting finding is that for each 1°C increment in temperature range (ΔT), the IEV increases by approximately 2%, which indicates a continuous reduction in bridge integrity throughout the country, if no intervention is conducted.

**Fig 7:**
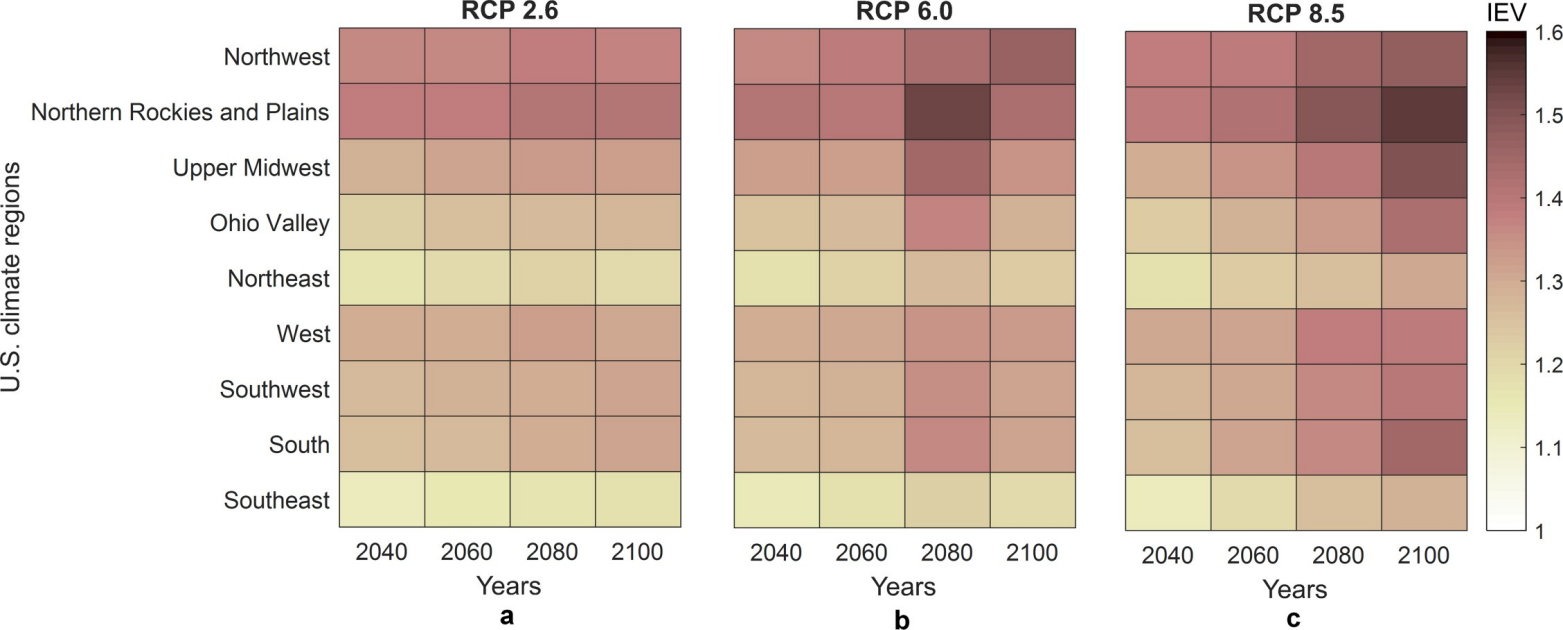
Variation of projected interaction equation value along the years for each U.S. climate region considering a) RCP 2.6, b) RCP 6.0 and c) RCP 8.5.

A regional analysis is carried out in order to assess the evolution of the average of interaction equation value over the years for U.S. bridges per climate region, as showed in Fig 7. This analysis considers the construction during the fall and compares the effect of the three different climate scenarios RCP 2.6, 6.0 and 8.5. Such analysis highlights the progressive increase of the undesirable thermal demand imposed on the bridges over the years when there is insufficient maintenance. The most critical regions, regardless the RCP, are the Northern Rockies & Plains, Northwest and Upper Midwest, while the less susceptible regions are the Southeast and Northeast. One should note that even considering different seasonal construction temperatures, the results are consistent as shown in Figure J in S1 Appendix.

**Fig 8:**
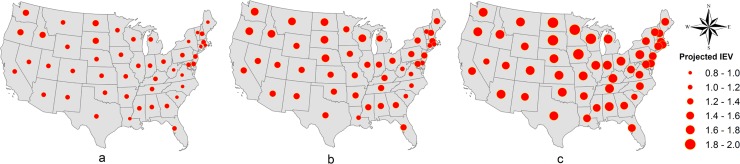
Ranges of interaction equation value by state for a) optimistic b) moderate and c) worst-case scenario.

Finally, the average IEVs are evaluated by state level for three hypothetical scenarios: optimistic–construction during the summer and projected temperatures under RCP 2.6; moderate–construction during the fall and projected temperatures under RCP 6.0; and the worst-case–construction during the winter and projected temperatures under RCP 8.5. This analysis considers the projected temperatures in 2100 (long-term analysis) and the results are spatially shown around the U.S. in Fig 8.

Regardless the hypothetical scenarios (a, b and c), the northern states present the highest IEV. For instance, one can note that South Dakota and North Dakota (Northern Rockies & Plains) are critical states for all the three scenarios.

## Conclusion

The impact of potential changes in temperature and clogging of joints on SSSG bridges in the U.S. is quantified, for the first time, by means of evaluating the interaction equation value for the main load carrying girders. In this study, we considered four different seasonal scenarios for bridge construction temperature, and three climate scenarios (RCP 2.6, 6.0 and 8.5) for projected temperatures for the years 2040, 2060, 2080 and 2100. Regardless the climate scenarios, the results of the analysis indicated that bridges located in the northern portion of the United States, such as Northern Rockies & Plains, Northwest and Upper Midwest are the most vulnerable due to the more pronounced increase in temperature ranges (difference between future and construction temperatures), although, the interaction equation value also depends on other parameters such as the length, geometry and materials properties of the bridge. Moreover, a state-level analysis, shows that South Dakota and North Dakota are the critical states (highest interaction equation values), regardless of the climate scenarios. In contrast, the less susceptible regions are the Southeast and Northeast. In addition, the analysis of the progressive increase in temperature along future years revealed that for each 1°C increment in temperature range (ΔT), the interaction equation value (IEV) increases approximately 2%, implying continuous reduction in bridge capacity as the climate warms if no intervention is conducted.

Neglecting possible future climate change can jeopardize the integrity of many U.S. bridges. Efficient maintenance to keep the expansion joints functional, on regular basis, is vital to avoid undesirable thermal demands that will potentially be magnified due to climate change. Nevertheless, given the vast number of deck joints bridges throughout the U.S., the challenge in maintaining the bridges cannot be overlooked. The presented analysis offers an overview from the current to expected future conditions of SSSG bridges. The aim is to rank the most critical bridges to be repaired amid an extensive inventory with approximately 89,000 structures. Such an approach can also offer subsidy for decision makers to prioritize the allocation of funds for structure maintenance and replacement. Further research is necessary to better establish a relationship between the interaction equation value and the level and characteristics of subsequent structural damage, which is critical for determining bridge functionality. This could be realized by first realizing the most critical bridges, then develop detailed computer models that can shed more light on local structural behavior in addition to global performance. Moreover, analysis should be conducted to quantify the expected life-cycle cost while accounting for social and economic losses associated with downtime corresponding to inspection, maintenance, repair, and failure. Such holistic analysis should account for uncertainties associated with all possible and relevant random variables including those associated with climate models.

## Materials and methods

### Temperature data

In order to evaluate the vulnerability of steel girder bridges due to induced thermal stresses that result from restrained movement caused by clogging of expansion joints, the corresponding temperature variation ΔT (°C) to which those structures will be exposed is calculated using Eq (1):

$$\Delta T=T-T_{o}$$ (1)

Where T is the projected temperature for future years (°C) and T_o_ is the base temperature (°C) at the stage of construction when the joints were installed. Thus, the superstructure of the bridge will expand from T_o_ to T (maximum temperature of exposition), and contracts from T_o_ to the minimum temperature of exposition. During instalation, and to ensure proper performance, when T_o_ is at mid range (average of maximum and minimum temperature that the bridge is subjected), the joint can be set at mid movement range. However, if T_o_ is above or below the mid range, the joint gap needs to be reduced or increased, respectively [38]. In this study, the temperature variation ΔT is considered constant along the bridge length and depth.

### Temperature during bridge construction

The temperature of each bridge construction at the stage in which the expansion joints were installed is estimated based on the year of bridge construction completion and the geographical position provided by NBI. First, we consider four possible scenarios for the construction of all bridges in the U.S.—construction during winter (Scenario 1), spring (Scenario 2), summer (Scenario 3) and fall (Scenario 4); thus, each bridge is assessed under all of these four possible scenarios. Each scenario can be interpreted as a hypothetical temperature condition, where Scenario 1 (winter) is the worst case–the range from low temperatures that occurs in the winter until projected daily maximum temperatures for future years provides the greatest amplitudes, Scenario 3 (summer) is the most optimistic–since this is the warmest season, the variation until the projected daily maximum temperatures for future years presents the smallest amplitudes, and Scenarios 2 and 4 have intermediate ranges of temperature. The State Code of each bridge, available in the NBI, is used to tie each bridge to one of the nine U.S. climate regions: Northwest, Northern Plains and Rockies, Upper Midwest, Ohio Valley, Northeast, West, Southwest, South, and Southeast. The temperatures for each bridge are then assigned for each scenario (for the respective year of construction conclusion) and are taken as the average of minimum temperatures for each season using the NOAA database of historical temperatures along the nine abovementioned U.S. climate regions [34] (see S1 Appendix). The option for taking the average of minimum temperatures (instead the average) is because it provides a larger temperature range to calculate the maximum thermal stress imposed on the structures. Bridges built prior to 1895 were not analyzed since temperature data for this period is not available.

### Projected temperature for future years (2040, 2060 2080, 2100)

The projected daily maximum temperature throughout the U.S. for years 2040, 2060, 2080 and 2100 are obtained from the NOAA GFDL CM3 coupled climate model that is part of the Coupled Model Intercomparison Project (CMIP5), under different Representative Concentration Pathway high-forcing scenarios (RCPs) [33]. The data with 1/8° resolution was downloaded from ftp://gdo-dcp.ucllnl.org/pub/dcp/archive/cmip5/bcca. MATLAB version R2018a was used to read and export the data into Arc Map version 10.5.1, where the projected daily maximum temperature of each bridge location for years 2040, 2060, 2080 and 2100 were extracted (see S1 Appendix). It is important to note that data for Alaska, Hawaii, and Puerto Rico were not available.

### Bridges data

Relevant bridge information presented in this research such as location along the U.S. highway, average age, status as deficient and obsolete, predominant type of design, length, among others (see S1 Appendix) was obtained from the 2017 National Bridge Inventory (NBI) from the U.S. Department of Transportation–Federal Highway Administration downloaded from https://www.fhwa.dot.gov/bridge/nbi/ascii.cfm.

**Fig 9:**
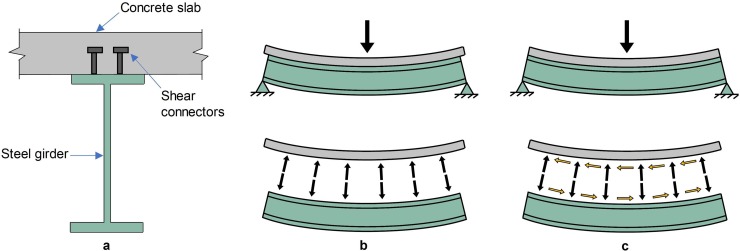
a) Steel-concrete composite section and a comparison between b) non-composite and c) composite steel-concrete beam.

The data for structural analysis was mainly obtained from the NBI; however, additional geometric information was estimated using the relationships given in [39,40] (see S1 Appendix). In addition, this study the superstructure of the bridges is comprised of steel-concrete composite I-girders due to their common use. The use of composite section in bridge construction began in the early 1930’s [35]. Furthermore, the adoption of composite steel systems became routine during the interstate era, with the construction of bridges after World War II [41]. Fig 9(A) illustrates a typical steel-concrete composite section while Fig 9(B) and 9(C) illustrates the difference in the behavior of a non-composite and a composite steel-concrete section, respectively. In the latter, there is no relative slip between the concrete slab and steel I-girder and consequently the entire cross section deflects as a single unit.

### Structural assessment

#### Interaction equation

Herein, the vulnerability of bridges with clogged joints that are subjected to temperature rise is quantified in terms of the interaction equation. A total of 89,089 simply supported steel girder bridges (SSSG) with compact section are evaluated. In this study we chose to evaluate only compact sections since the section capacity is governed by plastic behavior. The capacity of non-compact sections could have also been evaluated but would require much more information than what was readily available in the NBI database.

The interaction equation accounts for the demand-to-capacity ratio under axial loading and bending moment as given by Eq (2) and is a key design parameter for the main load carrying elements. As such, to ensure adequate structural performance of the bridge girders, this ratio should not exceed unity. Otherwise, the main girders will exceed their load carrying capacity. *It is critical to note that historically bridge girders are only evaluated for their moment capacity without the inclusion of the effect of the axial load because it is assumed that the expansion joints will always be functional*. *In the case of clogged joints*, *it is imperative to evaluate the bridge girders against their ability to not only carry bending moment but also the axial load as it interacts with the bending moment*. In this study, the bridges are analyzed under regular service conditions [39].

$$\frac{\gamma P}{\phi_{c}P_{n}}+\frac{\Sigma \gamma M}{\phi_{f}M_{n}}\leq 1$$ (2)

Where; P (kN) is the induced thermal axial compressive load when the superstructure of the bridge is restrained form expansion and M (kN.m) is the bending moment due to dead load (weight of the materials), live load (traffic) and the thermal load (due to the eccentricity of the thermal axial load); γ is the load factor for the limit state Service II [39]; P_n_ (kN) is the nominal axial compression strength and M_n_ (kN.m) is the moment strength of the composite superstructure of the bridge; ϕ_c_ and ϕ_f_ are the resistance factor for axial compression and flexure (ϕ_c_ = ϕ_f_ = 1 for service limit state [39]).

#### Restriction to the longitudinal expansion of the superstructure of the bridge

The effect of the clogged joints filled by sand and gravel, which restricts the longitudinal movement of expansion of the composite steel slab-concrete girder, is taken in account through the linear springs coefficients of the soil k_soil_ (*kN*/*m*) given by the Eq (3).

$$k_{soil}=\frac{E_{soil}A_{clogged}}{{\Delta L}_{joint}}$$ (3)

where; E_soil_ (*kN*/*m*^2^) is the modulus of elasticity of the soil, A_clogged_ (*m*^2^) is the cross sectional area of the joint clogged by debris and ΔL_*joint*_ (m) is the design thermal movement range of the bridge superstructure [39]. The modulus of elasticity of sand, gravel and the combination of sand and gravel is assumed respectively as 50,000 *kN*/*m*^2^, 150,000 *kN*/*m*^2^ and 100,000 *kN*/*m*^2^ [13]. Then, the effective stiffness k_eff_ (*kN*/*m*^2^) of the steel-concrete composite considering the stiffness of the soil is computed according to Eq (4).

$$k_{eff}={\left(\frac{1}{k_{composite}}+\frac{1}{k_{soil}}\right)}^{-1}$$ (4)

#### Thermal load

Once the expansion joints are clogged and the structure is exposed to a temperature variation, the superstructure is restrained from expanding. As a result, thermal stresses not predicted in the original design, are induced onto the girders and slab. The corresponding thermal load P (kN) that develops due to restraining of expansion of the superstructure, is accounted for in the first term of the interaction equation and can be computed according to Eq (5).

$$P=k_{eff}\varepsilon L$$ (5)

where; k_eff_ (kN/m) is the effective stiffness of the steel-concrete composite considering the effect of the stiffness of the soil according to the Eq (3), ε is the strain of the steel-concrete composite and L (m) is the length of the steel-concrete composite.

Since the steel girder and the concrete slab act as a unit, the strain in the concrete is equal to the strain in the steel. Thus, the actual strain of the steel-concrete composite ε is presented in Eqs (6) and (7).

$$\varepsilon =\alpha_{c}\Delta T+\frac{1}{E_{c}}\frac{\Delta T(\alpha_{s}-\alpha_{c})}{\left(\frac{1}{E_{c}}+\frac{A_{c}}{A_{s}E_{s}}\right)}$$ (6)

$$\varepsilon =\alpha_{s}\Delta T-\frac{1}{E_{s}}\frac{A_{c}}{A_{s}}\frac{\Delta T(\alpha_{s}-\alpha_{c})}{\left(\frac{1}{E_{c}}+\frac{A_{c}}{A_{s}E_{s}}\right)}$$ (7)

where; α_c_ and α_s_ (^o^C^-1^) are the coefficient of thermal expansion of concrete and steel; A_c_ and A_s_ (*m*^2^) are the cross sectional areas of the concrete slab and steel girder, respectively, and E_c_ and E_s_ are the modulus of elasticity (*kN*/*m*^2^) of the concrete and steel, respectively. In addition, the bending moment M_T_ (kN.m) due to the eccentric nature of the thermal axial load is calculated according to Eq (8).

$$M_{T}=P.e$$ (8)

where; P (kN) is the thermal load required to restrain expansion of the superstructure and e (m) is the distance from the point of application of P, that is, half of the depth of the clogged joint to the center of gravity of the composite section.

#### Resistances of materials

The commonly used concrete deck slab with 28-day compressive strength f′_c_ = 25 MPa [42] with 0.2 m is assumed. In addition, considering the evolution of the mechanical properties of the structural steel for bridges, the inventory is divided into three groups, in order to differentiate the steel strength: bridges built before 1901 with yield strength of 26 ksi, from 1901 to 1965 with yield strength of 36 ksi, and 1966 to 2017 with yield strength of 50 ksi [43,44].

#### Nominal moment strength of fully composite and compact sections *M*_*n*_

The calculation of M_n_ is based on plastic stress distribution on the composite sections. Tension in the concrete slab is neglected when the plastic neutral axis is in the slab since the concrete cannot carry tension.

#### Nominal axial compression strength of fully composite sections *P*_*n*_

The cross-sectional area of the concrete slab and the steel girder, and their respective resistances, compressive strength of the concrete– 0.85 f′_c_ (MPa) and yield stress of the steel–F_y_ (MPa), are considered for the contribution to the axial compressive strength of the composite P_n_.

## Code availability

All codes or algorithm will be made available to the editor and reviewers upon request.

## Supporting information

S1 Appendix (DOCX)
