# Supplementary material for: Impact of climate change on the integrity of the superstructure of deteriorated U.S. bridges
Source: PLoS One. 2019 Oct 23;14(10):e0223307. doi: 10.1371/journal.pone.0223307 (PMC6808546; doi:10.1371/journal.pone.0223307)
Supplement: S1 Appendix — (DOCX) [file pone.0223307.s001.docx]

**SUPPLEMENTARY INFORMATION**

**Impact of Climate Change on the Integrity of the Superstructure of Deteriorated U.S. Bridges**

**Susan Palu and Hussam Mahmoud**

1. **Bridges Data**
   1. ***Additional bridges analysis from National Bridge Inventory (NBI 2017)***

Figs A, B, C, and D below, obtained after processing NBI 2017 tabular data, show additional information related to simply supported steel girder (SSSG) bridges, which is the particular class of U.S. bridges assessed in this study. Fig A shows that the design type “Girder” is the most common on highways (which including Interstate, U.S., State and County highways). It corresponds to 245,957 out of 615,002 highway bridges in the nation (40%). In addition, this group has the largest number of functionally obsolete and structurally deficient bridges: 41,610 and 29,094, respectively.


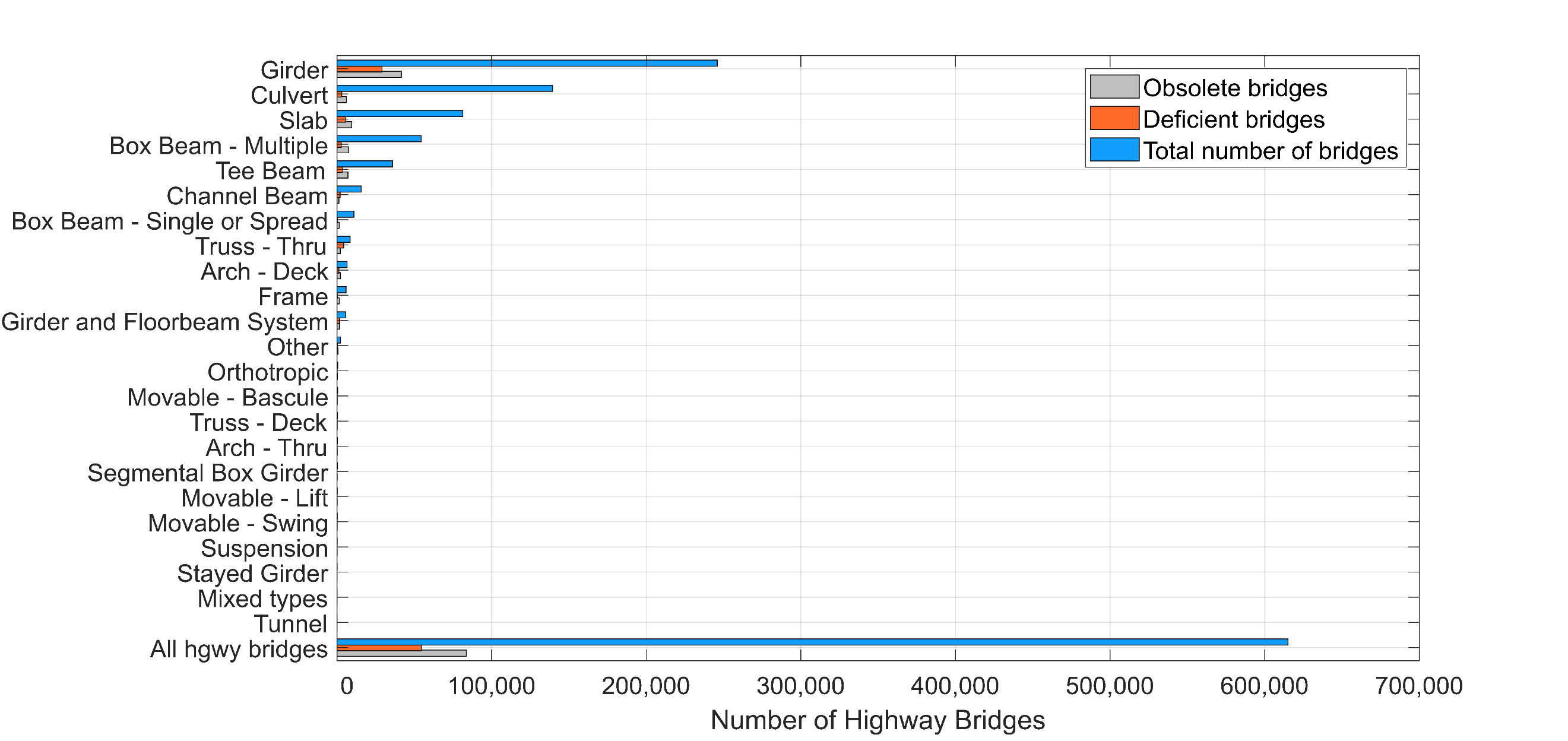


**Fig A. Distribution of U.S. highway bridges including all types of design.**

Furthermore, a close look into the deficient bridges of the country as presented in Fig B, show that girder-type bridges correspond to 53% of all deficient highway bridges, with the majority of them being SSSG (approximately one third of all highway bridges).

**Fig B. Pie chart of structurally deficient highway bridges.**

In addition, one can observe in Fig C that a significant number of girder bridges are among all types of bridges on Interstate highways, that carry the largest volume of the nation’s traffic. There are 33,587 girder-type bridges on interstates highways, which accounts for approximately 60% of the total.


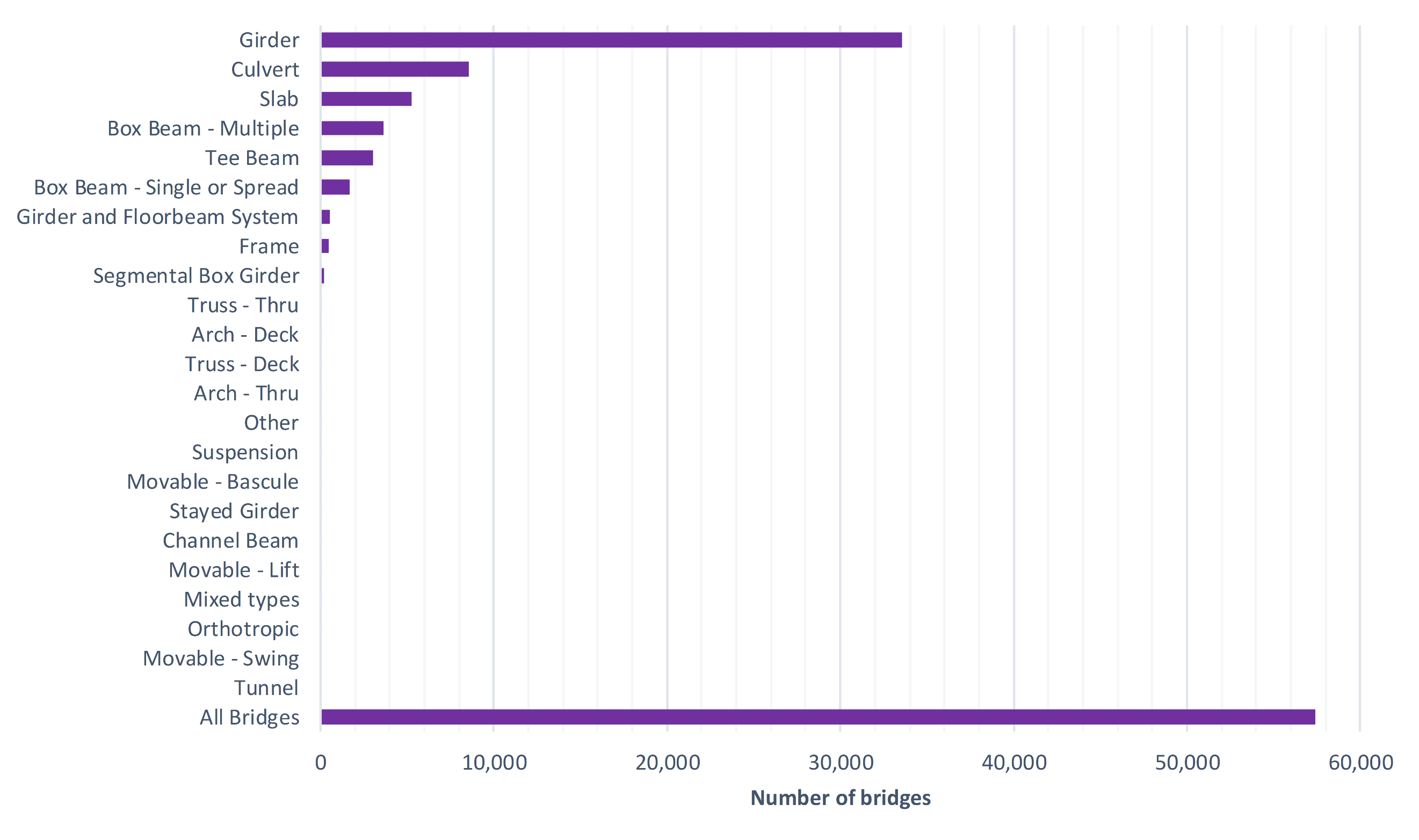


**Fig C. U.S. interstate highway bridges distribution including all types of design.**

Another important information processed from the tabular NBI inventory is the age distribution of the 97,393 SSSG bridges in U.S. From the pie chart in Fig D, one can observe that this particular class of bridges is aging, with more than half of structures exceeding 50 years in age. Indeed, the average age of 50 years is surpassing the national average bridge age of 45 years.


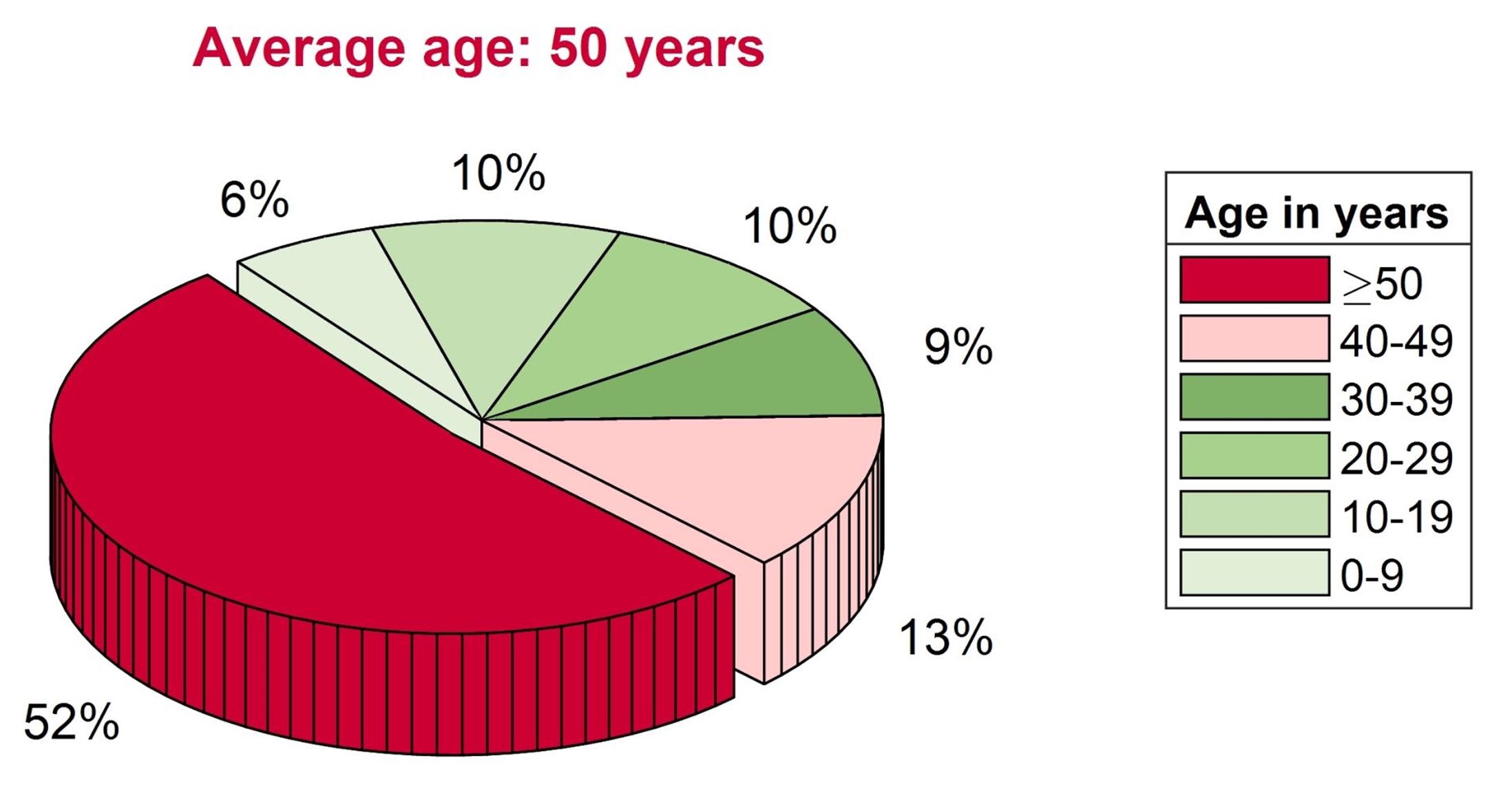


Fig D. Relative age distribution and average age of SSSG bridges in the U.S.

- 1. ***Estimate of girders geometry***

From the National Bridge Inventory data, the dimensions of the steel girder bridges could be estimated using the design equations in S.I. system. Initially, an approximation for girder depth $d$ (mm):

$$d=\frac{L}{24}\times1000$$

The weight per length is:

$$w_{t}=\frac{78.5M}{0.32F_{y}d}K$$

Where $w_{t}$ is the weight (kN/m), $M$ is the demanded bending moment (kN.m), $F_{y}$ is the steel yield stress (kN/m^2^), $d$ is the girder depth (m) and $K$ is a dimensionless reduction factor, approximately equal to 0.7 for composite sections (concrete and steel).

The cross-section area of the steel girder $A_{S}$ (mm^2^) is calculated as:

$$A_{S}=\frac{w_{t}}{78.5}\times1{000}^{2}$$

In addition, the moment of inertia $I_{x}$ (mm^4^) can be calculated as:

$$I_{x}=\frac{{0.16d^{2}w}_{t}}{78.5}\times1{000}^{4}$$

From the equation above one can obtain the elastic section modulus $S_{x}$ (m^3^)

$$S_{x}=\frac{{0.32dw}_{t}}{78.5}$$

1. **Temperature Data**
   1. ***Temperatures at bridge construction***

The temperature at the time of bridge construction is estimated based on the geographical position and the year of construction completion (data provided by NBI). Initially, the bridge location is associated with one of the nine U.S. climate regions: Northwest, Northern Plains and Rockies, Upper Midwest, Ohio Valley, Northeast, West, Southwest, South, or Southeast as shown in Fig E.


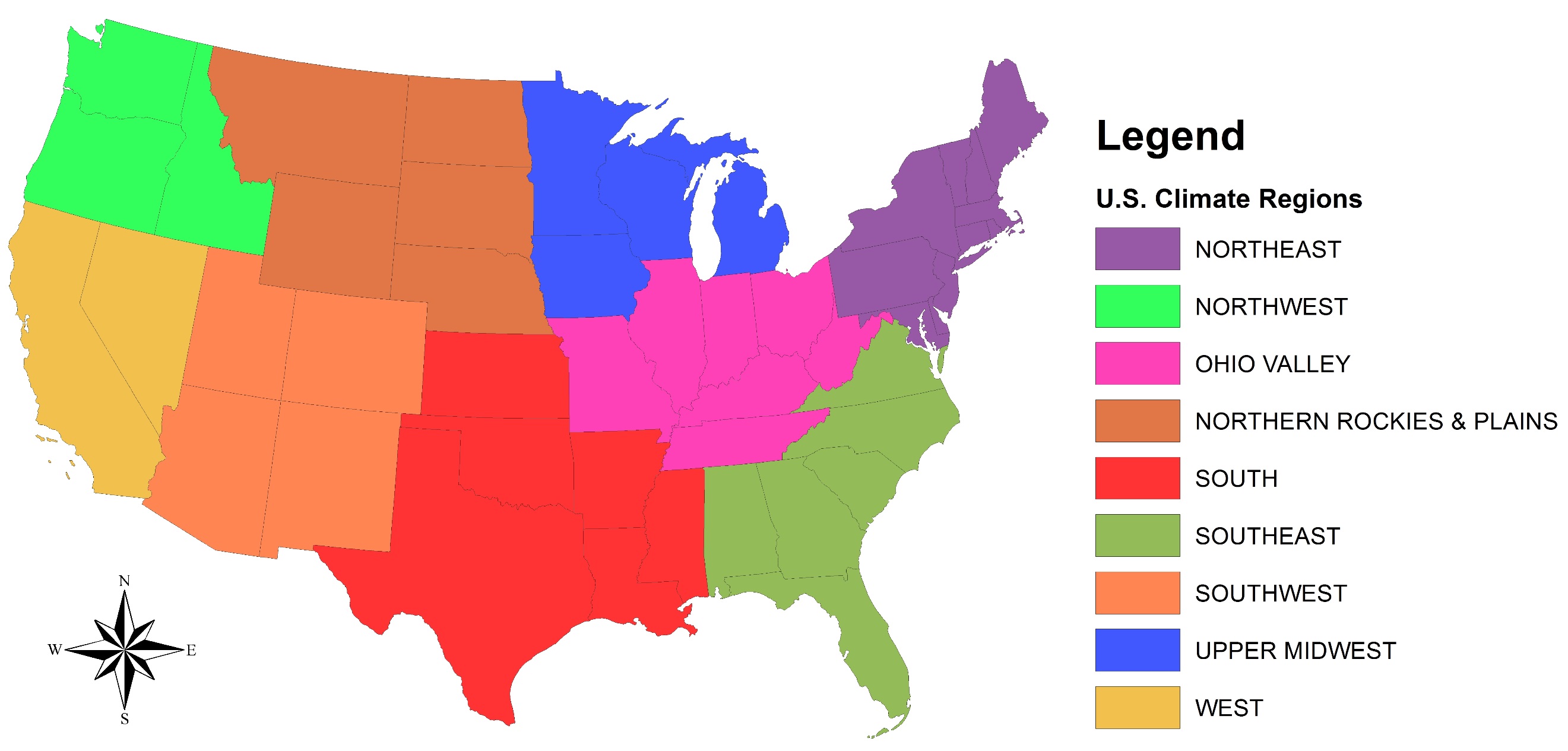


Fig E. U.S. climate regions.

NBI provides only the year of construction conclusion of the bridges. Thus, in order to assess uncertainty in the seasonal temperature variation, four scenarios are proposed: construction during winter (Scenario 1), spring (Scenario 2), summer (Scenario 3) and fall (Scenario 4).

The average temperature for each season of a specific year could be obtained from historical records along the U.S. climate regions from NOAA database (<https://www.ncdc.noaa.gov/cag/regional/time-series>). For the present analysis, the average minimum temperature for each season (and climate region) was used. The option for taking the average of minimum temperatures (instead the average) is because it provides a larger temperature range to calculate the maximum thermal stress into the structures in analysis. A summary of the historical temperatures for each of the nine U.S. climate regions is illustrated in Fig F.


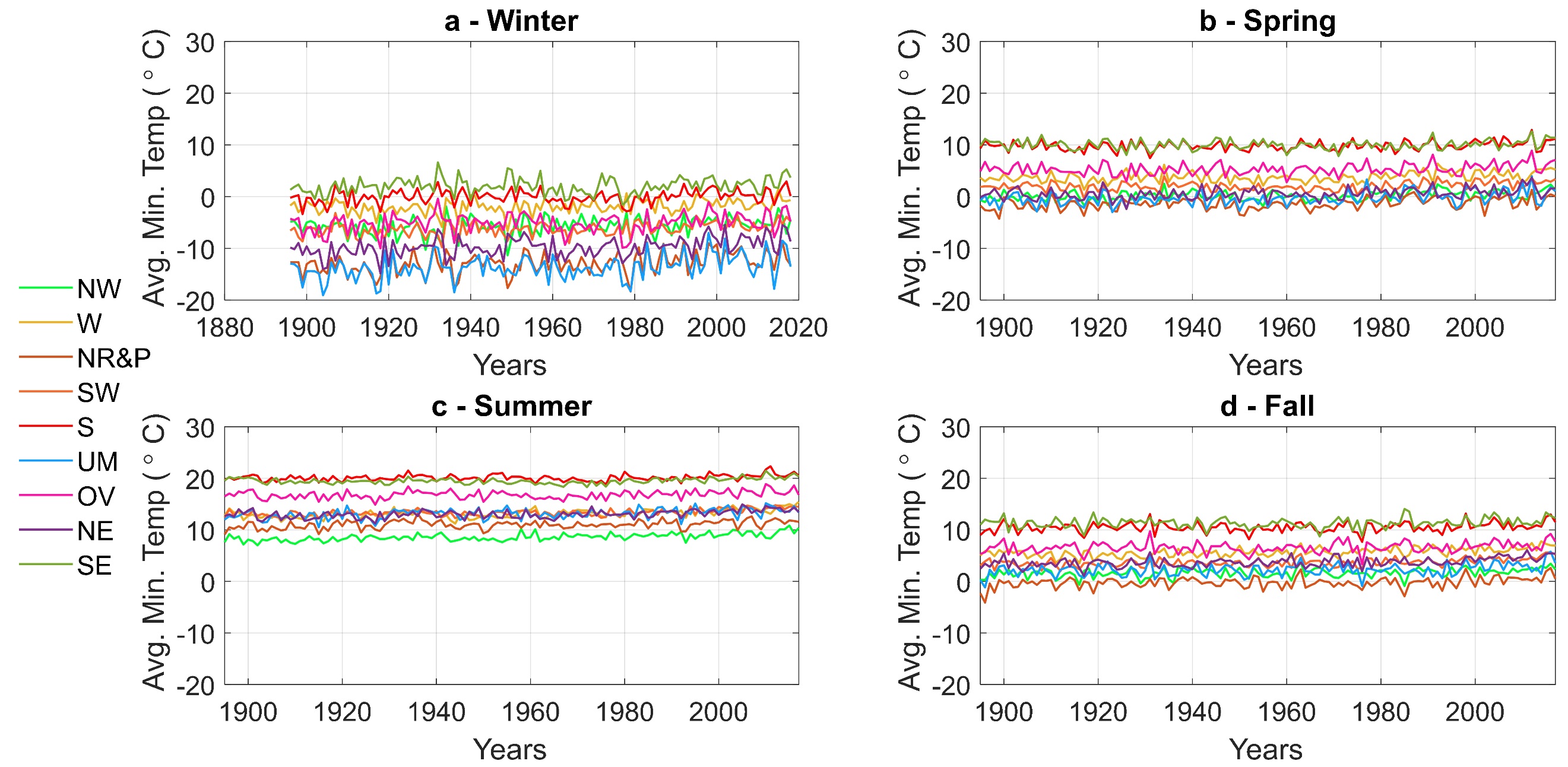


**Fig F. Historical average minimum temperature along the years for each region evaluated in winter, spring, summer and fall.**

Table A presents an example of historical temperature data processing procedure in order to account for the temperature at the construction (i.e. onset of installation of joint) of a specific bridge.

**Table A. Example of historical temperature data processing procedure to account for the temperature at construction for bridge B-16-FM**

| Data Available in NBI | | | Data Processed | | | | |
| --- | --- | --- | --- | --- | --- | --- | --- |
| State Code | Structure Number | Year  Built | U.S.  Climate Region | Avg. Min. Temp. (°C)  Winter  Scenario 1 | Avg. Min. Temp. (°C)  Spring  Scenario 2 | Avg. Min. Temp. (°C)  Summer  Scenario 3 | Avg. Min. Temp. (°C)  Fall  Scenario 4 |
| 8 | B-16-FM | 1966 | Southwest | -7 | 2 | 14 | 4 |

The first three columns in Table A represents the bridge data provided by NBI. The State Code is given by item 1 of NBI and corresponds to the Federal Information Processing Standards (FIPS) code for States. The Structure Number is given in item 8 of NBI and it is a unique code for each bridge. The Year Built is listed as item 27 in the NBI data and it represents the year of construction completion. For this example, the bridge identified as B-16-FM is located in Colorado State (FIPS code 8) and had its construction completed in 1966. With that information, this particular bridge is classified to belong to the Southwest climate region and the average minimum temperature of each season for the year 1966 (shown in Table A) is extracted from the historical records from NOAA database. Therefore, the four scenarios that simulates the construction of the bridge during each season can be evaluated.

- 1. ***Projected temperature for future years***

The projected daily maximum temperature throughout the U.S. for years 2020, 2040, 2060, 2080 and 2100 are obtained from GFDL CM3 coupled climate model from the National Oceanic Atmospheric Administration (NOAA) Geophysical Fluid Dynamics Laboratory, which is part of the Coupled Model Intercomparison Project (CMIP5). The present analysis accounts for the Representative Concentration Pathway including low-forcing scenario RCP 2.6 (the most optimistic emissions scenario), moderate scenario RCP 6.0 and high-forcing scenario RCP 8.5 (the most unfavorable emissions scenario). The data with the highest resolution (1/8°) was downloaded from <ftp://gdo-dcp.ucllnl.org/pub/dcp/archive/cmip5/bcca>.

The abovementioned data provides the projected daily maximum temperature for approximately seventy-six thousand geographic coordinates along the U.S. main territory and it is available in blocks of five years. Thus, five blocks of data were processed: 2016-2020, 2036-2040, 2056-2060, 2076-2080 and 2096-2100, for each RCP. MATLAB version R2018a was used to read and process these temperature data. As a result, a daily maximum temperature grid was generated for future years 2020, 2040, 2060, 2080, 2100. The points of the grid take the highest daily temperature reached during the 5 years interval. Then, the grids of daily maximum temperature for 2020, 2040, 2060, 2080 and 2100 were processed in ArcMap version 10.5.1. The respective maps are illustrated in Fig G.

.


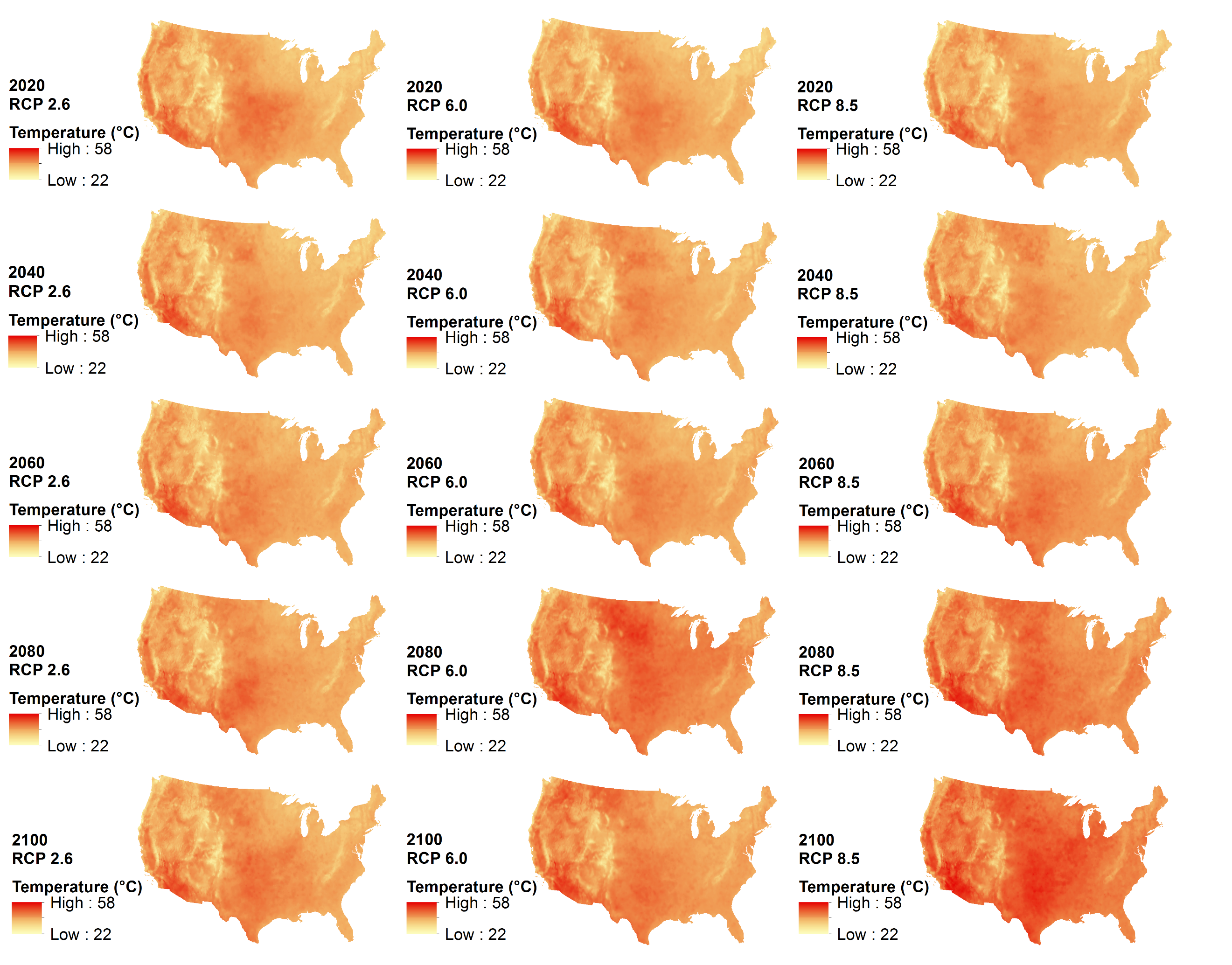


**Fig G. Projected daily maximum temperature from NOAA climate model GFDL CM3 for a) RCP 2.6, b) RCP 6.0 and c) RCP 8.5.**

After combining the geographical temperature information with the location of the bridges (available in NBI 2107), the extraction of the projected daily maximum temperature for future years of each one of the 89,089 bridges under analysis was possible. As example to illustrate this procedure, in Fig H, one can see the projected temperatures for 2100 (under the higher forcing scenario RCP 8.5) and all simply supported steel girder bridges (SSSG bridges). These future temperatures (in association with the temperatures during bridge construction) are the temperature inputs to assess the aggravated effect of clogged joint.


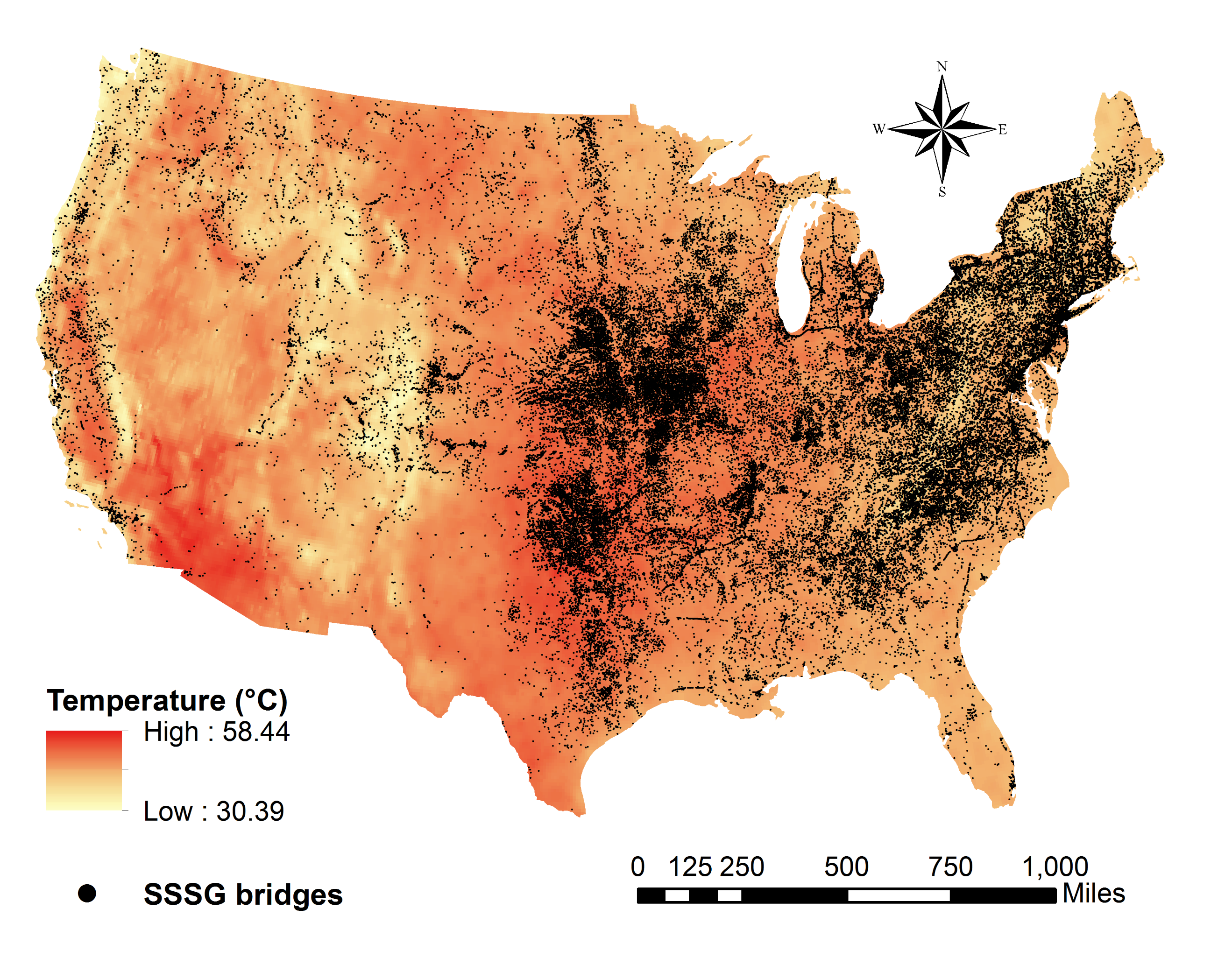


Fig H. Projected daily maximum temperature for 2100 (under the higher forcing scenario RCP 8.5) and location of SSSG bridges.


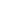

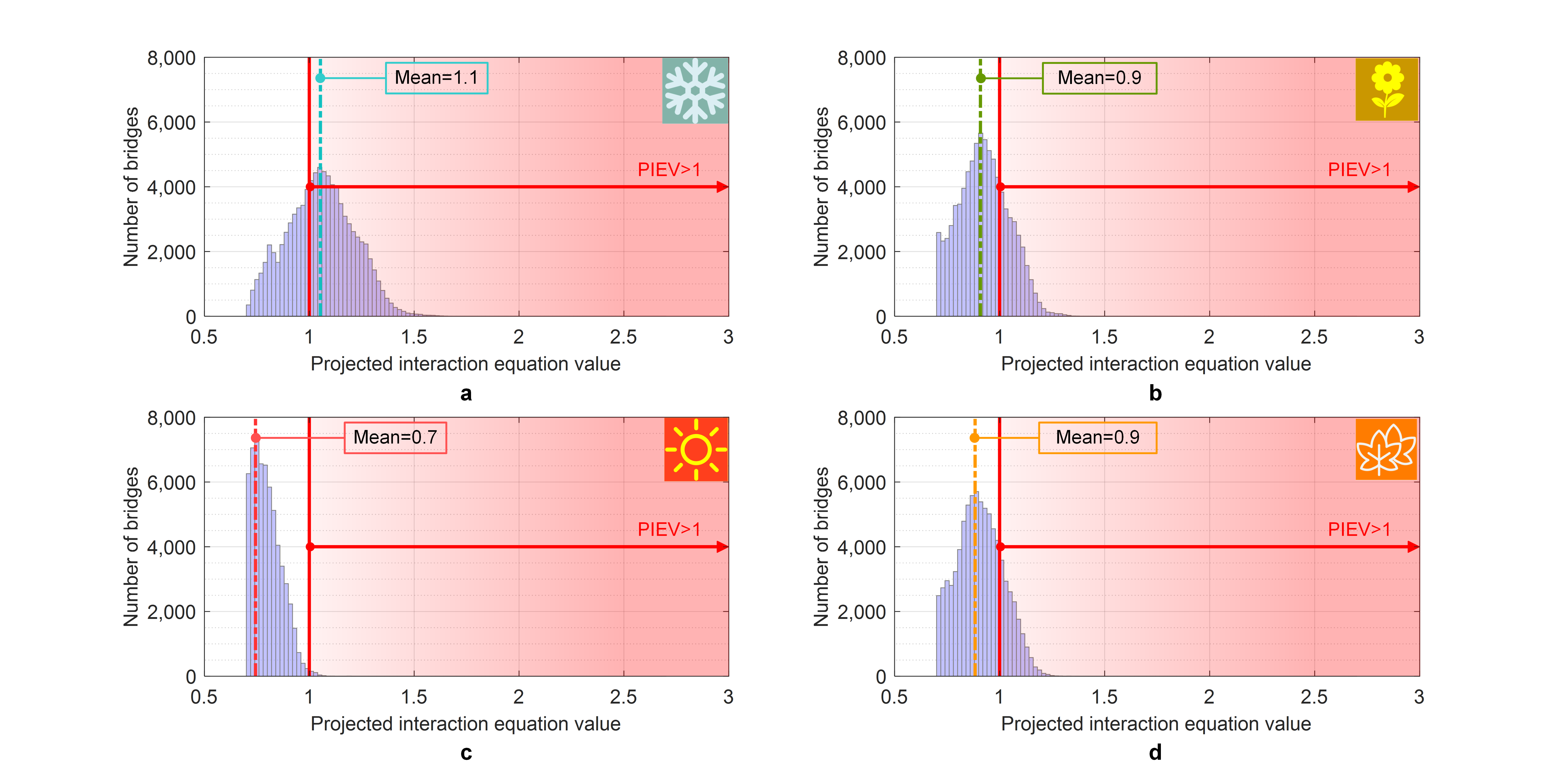


Fig I. Histograms of the calibrated and projected interaction equation value (PIEV) for temperatures in 2100 (under the high forcing scenario RCP 8.5 considering a) Scenario 1, b) Scenario 2, c) Scenario 3 and d) Scenario 4.

The most critical regions, in a descending order, are: Upper Midwest and Northern Rockies & Plains for the Scenario 1 (winter), Northern Rockies & Plains and Northwest for the Scenario 2 (spring), Northwest and Northern Rockies & Plains for the Scenario 3 (summer), and Northern Rockies & Plains and Northwest for the Scenario 4 (fall). Moreover, regardless of the scenario adopted, the Southeast is the region less impacted. Furthermore, it is important to highlight that the average interaction value in all regions is above one, except the Northeast for the year 2040 and the Southeast for the years 2040 and 2060.


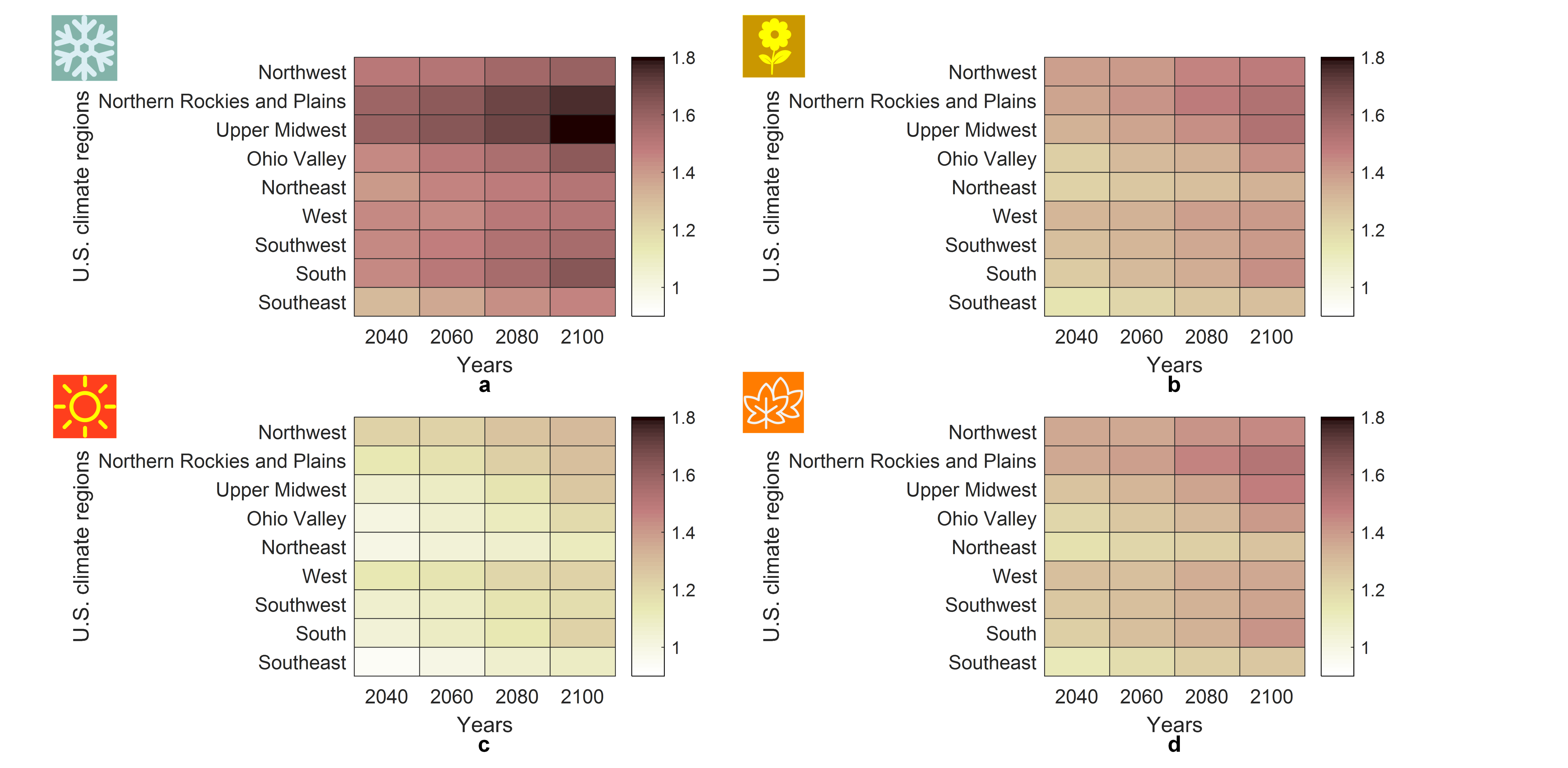


Fig J. Variation of projected interaction equation value along the years for each U.S. climate region considering a) Scenario 1, b) Scenario 2, c) Scenario 3 and d) Scenario 4.
